# The right not to know and the obligation to know

**DOI:** 10.1136/medethics-2019-106009

**Published:** 2020-04-29

**Authors:** Ben Davies

**Affiliations:** Uehiro Centre for Practical Ethics, University of Oxford, Oxford, UK

**Keywords:** informed consent, autonomy

## Abstract

There is significant controversy over whether patients have a ‘right not to know’ information relevant to their health. Some arguments for limiting such a right appeal to potential burdens on others that a patient’s avoidable ignorance might generate. This paper develops this argument by extending it to cases where refusal of relevant information may generate greater demands on a publicly funded healthcare system. In such cases, patients may have an ‘obligation to know’. However, we cannot infer from the fact that a patient has an obligation to know that she does not also have a right not to know. The right not to know is held against medical professionals at a formal institutional level. We have reason to protect patients’ control over the information that they receive, even if in individual instances patients exercise this control in ways that violate obligations.

It is widely believed^1–4^ that doctors require very strong reasons if they are to withhold information from patients that is relevant to their health and care. In other words, as a patient I have a Right to Know information that is relevant to my health. Somewhat more contentious is the claim that I have a corresponding claim to medical ignorance, that is, a Right Not to Know (RNTK).

As generally understood, both the Right to Know and the RNTK will not qualify as genuine rights on some analyses of that term. McDougall notes that, although they may commonly be presented as such, it is difficult to defend the relevant claims as ‘prioritised non-outweighable’ interests, which cannot be overridden in any circumstances.^5^ In other words, it cannot be that one has either a claim to have or reject information that applies *in all* circumstances *no matter what* the costs. Instead, I will understand both purported rights as claims to which significant priority must be given, and which can only be overridden by certain kinds of equally weighty claims. While rights in the relevant sense may ‘trump’ less significant interests, they need not be absolute.^6^

Two central arguments for a RNTK are the appeal to harm and the appeal to autonomy.^1^ With respect to harm, the thought is that sometimes medical news can be extremely distressing, while diagnosis of other conditions can lead to social stigma and discrimination.^7–9^ Particularly where there is no effective cure, receiving a diagnosis may simply not be worth it for the patient. Opponents of the RNTK object that in many cases, the harms involved in receiving unwanted medical knowledge are ‘transient and mild’.^10–12^

The appeal to autonomy relies on the idea that an autonomous life is guided by an agent’s own choices.^13–17^ A person should therefore not be forced to receive information she does not want because that would mean she was not directing her own life.

Arguments against the RNTK also invoke autonomy, harm to others and in some cases harm to the patient.^12 18^ With respect to autonomy, Harris and Keywood argue that even if a patient wants to avoid certain information about themselves, an appeal to autonomy cannot support this because autonomous decision-making requires relevant information.^19^ Similarly, Rhodes argues that refusing to hear relevant information amounts to leaving matters to chance, and that ‘if autonomy is the ground for my right to determine my own course, it cannot also be the ground for not determining my own course’.^20^ ^2^ A related argument concerns the rights and obligations of medical professionals; Hull rejects a RNTK on the grounds that knowledge is necessary for informed consent, and that without informed consent, certain medical procedures would constitute assault.^21^

The RNTK has been challenged more specifically with respect to genetic information on the grounds that the decision about whether to hear medically relevant information does not only affect the individual patient. Since genes are shared among family members, genetic information about myself is also (probabilistic) genetic information about my biological family.^22–28^ ^3^ If I have information about myself that is also inherently information about my family, I may have an obligation both to hear it and to pass it on.

This latter argument relies on harms to others as the grounds for rejecting a RNTK. It is therefore not one that can be immediately refuted by an appeal to autonomy. For even if we think that a patient’s autonomy automatically rules out giving them information that they do not want for *paternalistic* reasons, that is, for reasons of their own good, it cannot perform the same role with respect to harms to others. In the existing literature on the RNTK, we can also find some brief references to rejections in other cases that are based on harm to third parties. Harris and Keywood imagine an individual who does not wish to know their HIV-status but does wish to carry on being sexually active.^19^ Kielstein and Sass consider a ‘duty to know’ in the context of being a responsible parent.^29^ Finally, the Council of Europe’s discussion of rights in biomedicine suggests that the various rights outlined—including a RNTK—may be limited in cases where this is necessary for ‘public safety, for the prevention of crime, for the protection of public health or for the protection of the rights and freedoms of others’.^1^ In all cases, the RNTK is at least bounded by the likelihood that ignorance will involve passing on costs to others.

Such an argument may take two forms. The stronger form argues that once suitably strong interests of other people besides the patient are involved, the patient no longer has a claim against their doctor (or other medical professionals) not to tell them relevant information. They may even lose the right that their doctor does not tell *others*, that is, those affected, the patient’s normally private medical information. The weaker form of this argument is that while medical professionals still have obligations to their patients to respect their wishes not to know, it is no longer morally innocent of the patient to *enforce* that right. On this view, while the patient still has the RNTK in an *institutional* context, they do not have such a right at a more general moral level. I will say more about this idea of rights operating at different contexts shortly.

Arguments against the RNTK that appeal to the interests of those besides the patient typically focus on harms to particular individuals who have some connection to the patient (eg, existing family; possible future children; sexual partners). Some authors do also consider the broader social good. Rhodes supports a duty to participate in population studies,^20^ while Knoppers and Chadwick note that such participation might be supported by an appeal to solidarity.^30^

But it is also possible to argue for a defeasible moral obligation to acquire relevant knowledge about one’s health—and, thus, the absence of a moral right not to do so—in a different way. This argument appeals to a further obligation: not to impose unreasonable, avoidable burdens on others. In a pluralistic, liberal society, individuals should have considerable freedom to pursue the kind of life that they consider best. Such pursuit, according to classical liberalism, should be constrained neither by appeal to either the individual’s own welfare, nor the purported irrationality of their values.

However, this position does not rule out all *moral* constraints on individual citizens in their pursuit of the good life. In particular, we may still hold that in our free actions, individual citizens have an obligation to reduce where reasonable the costs to others in society. If I face two possible ways of pursuing my goals, and one involves imposing greater burdens on the rest of society, it is consistent with liberal political values to say that I ought to choose the less burdensome option. Note that the argument is importantly *not* that anyone has the right to *force* me to pursue the less burdensome method of pursuing my reasonable goals. As I detail further on, there are many things that I have a moral obligation to do or refrain from doing, but which nobody has a right to force me to do, or coercively prevent me from doing. For instance, I ought not knowingly give misleading directions to tourists, and I ought not snap at innocent questions from my children just because I have had a bad day. But while people might morally condemn or chastise me for these behaviours, nobody ought to force me to act differently.

In some cases, patients who refuse relevant medical information will make their health problems worse, more complicated and more expensive to treat. If such patients live in a society that provides publicly funded healthcare, and they make a claim for such care (as they have every right to do), then their initial refusal of information has increased the overall costs to the rest of society. When their refusal of information was unreasonable (I discuss this below), then they have violated a moral obligation not to unreasonably impose burdens on others. However, this does not mean that anyone is entitled to *force* them to hear the relevant information. While there might be some levels of burden that would justify this latter choice, there is no reason to suppose that the two levels—that is, the degree of burden at which one has a moral obligation not to impose it, and the degree at which others have a right to *stop* you imposing it—are the same.

Some clarifications are required. First, this argument applies most clearly to a publicly funded health system, where financial burdens do not fall directly on the patient. A patient who pays for their own care may act unwisely in refusing relevant information but may not act immorally (at least in virtue of the current argument). However, it is important to note that this is not straightforward. For instance, actions in the private healthcare market may have effects on a parallel, public system (eg, by using up resources such as the working time of medical professionals, or scarce medicines). Moreover, there may be parallel obligations within private systems to others in that system. Finally, in many countries even patients who pay for private medical care have access to publicly funded care in some forms. For instance, in an emergency, we do not check whether the patient is a public or private patient before calling a publicly funded ambulance, for obvious reasons.

Second, it is worth acknowledging that the problematic aspect of exercising the RNTK is, strictly speaking, in the predictable choices that follow, that is, choices that make one’s health worse or more difficult to treat. In some cases, patients might be able to avoid imposing unreasonable burdens on others by following medical advice despite remaining ignorant. But this will not always be possible; some medical advice that would alleviate a particular condition might be contraindicated for a patient who did not have that condition. In such cases, the very fact that certain advice was given would tell a patient what they did not want to know.

Finally, the argument applies only to the imposition of *unreasonable* burdens. My example above involved a case where the same good could be pursued in more or less burdensome ways. But in other cases, it may be necessary to make a choice that will burden others more, in order to pursue something that one could not otherwise acquire, or to avoid a serious harm. My argument thus recognises that there are cases, perhaps many, where the additional health, and cost, burdens that stem from choosing not to know may be a *reasonable* choice when compared with the alternative.^31–40^ The relevant obligation is not, importantly, an obligation to minimise the demands one makes on the healthcare system. In addition, the argument applies only to cases where ignorance would lead to a greater overall health burden, that is, cases where the medical condition can be successfully treated or mitigated. Individuals who refuse information about their health status with respect to conditions that cannot be treated may fail other obligations (eg, to their close relatives), but do not thereby fail an obligation not to impose greater burdens on the health system.

I suggest, therefore, that in addition to existing objections to an absolute RNTK, we may add cases where choosing to remain ignorant would impose unreasonable costs on the rest of society.

If we do have an obligation to know in such circumstances, what does this imply about a RNTK? On first glance, it might seem that the answer is straightforward. One might think similarly to Rhodes who, discussing specifically genetic conditions, writes that ‘if someone has a right to genetic ignorance, he has no duty to pursue genetic knowledge, and if someone has an obligation to pursue genetic knowledge, she has no right to preserve her genetic ignorance’.^20^ This is supposed to follow directly from the meaning of the terms ‘right’ and ‘obligation’. So, it might seem that in cases where we have an obligation to receive information, we cannot also have a RNTK that same information.

But the RNTK is not so straightforward as this argument implies.^14 41 42^ For the right is typically discussed and claimed in the context of the relationship(s) between patient and medical professionals. As such, the RNTK is, in practice, a right ‘not to be told’ unwanted information, typically held against medical professionals.^43 44^

I emphasise once more the difference between the existence of a moral obligation per se, and the existence of an obligation that others have a right, or even a duty, to enforce. The obligation I have proposed is that one not impose unreasonable burdens on others. But one might accept that we all have such an obligation yet note that it is often difficult to tell whether someone satisfies the relevant criterion of reasonableness. A doctor who is confronted by a patient who says that he does not wish to receive medically relevant information does not know—at least initially—whether this refusal is reasonable or not. As such, even if the patient genuinely is violating an obligation, it may be impermissible for the doctor to enforce the putative obligation by telling the patient the relevant information. It may even be impermissible for her to *moralise* to the patient by trying to make him feel guilty. For even if the patient *would* violate an obligation were his refusal of information to be unreasonable, the burden placed on others is not sufficiently significant to warrant the risk of placing pressure on someone who has a legitimate reason for avoiding medical information.

This raises a further point. Whether a burden is reasonable depends in part on whether it is foreseeable. A patient who does not know anything about her health status may not be in a position to foresee the risks of refusing information. There is, therefore, something almost paradoxical about the RNTK, where a *minimal degree* of information (ie, a sufficient amount to know that refusing further information would be risky) generates an obligation to acquire further information, whereas being less informed would generate no such obligation. We might avoid the air of paradox by suggesting that even if medical professionals ought not moralise to their patients, and even if they ought to respect their wishes to remain uninformed, they may still have an obligation to remind patients *in general terms* of the potential risks of refusing medically relevant information.^45^

Even if a member of a patients’ medical team were somehow to be in an idealised position to judge her patients’ reasons, it is consistent with a patient’s having an obligation to acquire information that they have a right not to be told that information. For it is not the medical professionals’ task to enforce all of their patient’s moral obligations, even those that arise in a medical context. This way of thinking about the RNTK sees the respective obligation and right as operating at different levels, and for different reasons. For instance, if it is true that, as I have argued, an obligation to know medically relevant facts can be derived from a more fundamental obligation not to impose unreasonable burdens on one’s fellow citizens, then this obligation may be seen as operating at a fundamental moral–political level. But this is consistent with there being a right at the institutional–legal level not to know (or, more strictly, not to be told) medically relevant information, a ‘right to do wrong’, which may be justified quite differently.^46^ For instance, it may not be that I have a basic moral claim on remaining ignorant of information that would help me fulfil other obligations, but rather that I have a moral claim on people in positions of social and institutional power (such as medical professionals) respecting my preferences about how they treat me. This is consistent with it being wrong of me to ask that I am treated in that way. It is thus overly simplistic to point to a (basic moral) obligation to know as evidence against an (institutional) RNTK.

Waldron describes the claim that I can have a legal right to perform a moral wrong as uncontroversial. As such, it is important to note that I am making a slightly stronger claim here: that is, I am not only stating the truism that I might have a legal right (due to its presence in the law of the country I live in) to do something that is wrong. Rather, I am agreeing with Waldron’s stronger claim, that it can be *morally best* to set up such legal protections against people being forced to do what is right. As Waldron notes, we can understand the claim that ‘P has a moral right to do A’ as implying that ‘It is morally wrong for anyone to interfere with P’s doing A’, which is compatible with ‘P’s doing A is morally wrong’.^46^

For instance, consider what might happen if Dr Walker discovers that his patient Joe is infertile, and Joe plans not to tell his wife, Beth. Beth wants children, but Joe does not want to pay for in viro fertilisation, and so will persuade his wife to keep trying to conceive through sexual intercourse.

What Joe does is wrong. He has an obligation to tell his wife the truth. But this does not imply an obligation or permission for *Dr Walker* to force him to fulfil that obligation. I do not suggest that such cases will always be simple; if Beth is also a patient of Dr Walker’s, he may have conflicting obligations.^47^ My point is simply that even quite significant obligations do not imply a lack of rights; Joe has a right that his doctor respects his privacy, even if that facilitates him behaving immorally.

Similarly, an amended version of Waldron’s analysis can be applied to the case of the RNTK. First, I have suggested that the RNTK is held only against medical professionals. It need not, for instance, imply that it is wrong of my father to inform me of genetic information about himself, thereby indirectly informing me of information about *my*self. As such, we may say that I have an RNTK *held against medical professionals*, which implies, to adapt Waldron’s principle, that ‘it is morally wrong *for medical professionals* to interfere with my choosing to remain ignorant about my health status’. This is compatible with that choice being wrong. Second, as I stated at the beginning of this paper, we need not understand such a right as absolute. So, we may wish to add, for instance, that it is morally wrong for medical professionals to interfere ‘except in circumstances where doing so is required to avoid serious harm to others’.

Even if a patient is in a situation where it would be wrong of them to fail to seek out relevant information about their health, they may nonetheless have a legitimate claim against medical professionals that they not be given that information unless they consent to receive it. As Waldron notes, though, while such a right may imply an obligation on the part of others not to interfere, that obligation may not itself be absolute. For instance, your right to be rude to people serving you in restaurants implies that I may not aggressively coerce you to politeness; but it does not imply that I may not chastise, criticise or attempt to reason with you. Similarly, a RNTK may be consistent with it being permissible for medical professionals to point out the potential negative effects of remaining ignorant. Nonetheless, this must be approached carefully; for instance, since I have suggested that the obligation to know depends on knowledge being necessary to avoid imposing *unreasonable* burdens on others, medical professionals should be confident that their patient lacks good reason to remain ignorant before attempting to persuade them to do the right thing. The difficulty of such a task may make it pragmatically best to err on the side of caution in most or even all cases.

Opposition to the RNTK in practice, then, cannot derive solely from the claim that patients often have an obligation to acquire knowledge about their condition. Patients may have an obligation to know, and yet a right, held against medical professionals, not to be told information that they do not want to receive.

## Footnotes

An alternative grounding rests on our interest in privacy. Laurie argues that unsolicited information necessarily encroaches on private space which has “presumptive inviolability”.^48^ See also Herring and Foster.^13^

See also.^12 49 50^ Bortolotti^11^ offers a critical discussion of this view.

See^2 41 51 52^ for critical discussion.
